# Molecular Detection and Characterization of the First Cowpox Virus Isolate Derived from a Bank Vole

**DOI:** 10.3390/v11111075

**Published:** 2019-11-18

**Authors:** Kathrin Jeske, Saskia Weber, Florian Pfaff, Christian Imholt, Jens Jacob, Martin Beer, Rainer G. Ulrich, Donata Hoffmann

**Affiliations:** 1 Institute of Novel and Emerging Infectious Diseases, Friedrich-Loeffler-Institut, Federal Research Institute for Animal Health, Südufer 10, 17493 Greifswald-Insel Riems, Germany; kathrin.jeske@fli.de (K.J.); rainer.ulrich@fli.de (R.G.U.); 2 Institute of Diagnostic Virology, Friedrich-Loeffler-Institut, Federal Research Institute for Animal Health, Südufer 10, 17493 Greifswald-Insel Riems, Germany; saskia.weber@fli.de (S.W.); florian.pfaff@fli.de (F.P.); martin.beer@fli.de (M.B.); 3 Vertebrate Research, Institute for Plant Protection in Horticulture and Forests, Julius Kühn-Institute, Toppheideweg 88, 48161 Münster, Germany; christian.imholt@julius-kuehn.de (C.I.); jens.jacob@julius-kuehn.de (J.J.)

**Keywords:** bank vole, *Myodes glareolus*, cowpox virus, orthopoxvirus, Germany

## Abstract

*Cowpox virus* (CPXV) is a zoonotic *orthopoxvirus* (OPV) that infects a wide range of mammals. CPXV-specific DNA and antibodies were detected in different vole species, such as common voles (*Microtus arvalis*) and bank voles (*Myodes glareolus*). Therefore, voles are the putative main reservoir host of CPXV. However, CPXV was up to now only isolated from common voles. Here we report the detection and isolation of a bank vole-derived CPXV strain (GerMygEK 938/17) resulting from a large-scale screening of bank voles collected in Thuringia, Germany, during 2017 and 2018. Phylogenetic analysis using the complete viral genome sequence indicated a high similarity of the novel strain to CPXV clade 3 and to OPV “Abatino” but also to *Ectromelia* *virus* (ECTV) strains. Phenotypic characterization of CPXV GerMygEK 938/17 using inoculation of embryonated chicken eggs displayed hemorrhagic pock lesions on the chorioallantoic membrane that are typical for CPXV but not for ECTV. CPXV GerMygEK 938/17 replicated in vole-derived kidney cell lines but at lower level than on Vero76 cell line. In conclusion, the first bank vole-derived CPXV isolate provides new insights into the genetic variability of CPXV in the putative reservoir host and is a valuable tool for further studies about CPXV-host interaction and molecular evolution of OPV.

## 1. Introduction

Members of the genus *Orthopoxvirus* (OPV) belong to the subfamily *Chordopoxvirinae* within the family *Poxviridae.* The relatively large enveloped virions are generally brick-shaped and contain a double-stranded linear DNA genome. The viral genome consists of a unique region that is flanked by inverted terminal repeats (ITR). While the core genome, encoding proteins that are essential for the viral DNA replication machinery as well as structural and regulatory factors, is highly conserved, the flanking and ITR regions encode host response modulating proteins that vary between different OPV species [1]. Currently, the genus *Orthopoxvirus* comprises ten species [2]. These virus species differ drastically in their host range: Although the infection with *Variola virus* (VARV), the eradicated causative agent of smallpox, was limited to humans, some other OPV do cross species barriers. For example, *Monkeypox virus* (MPXV), *Vaccinia virus* (VACV), and *Cowpox virus* (CPXV) have a wide host range and can cause spillover infections in multiple non-reservoir species [3,4]. Zoonotic CPXV is endemic in Eurasia and believed to be a “rodent-borne” virus. CPXV-related disease has been reported from 27 host species, including humans, cats, livestock, and zoo animals [5,6]. Most of these mammals need to be considered accidental dead-end rather than reservoir hosts, as maintenance of CPXV in these species does not occur. The broad host range of CPXV is thought to be mediated by a large number of genes, resulting in the most multitudinous genetic repertoire of all known OPV [7,8]. CPXV spillover infections from animals to non-vaccinated persons usually result in local skin lesions but rarely cause generalized and fatal disease in immunocompromised patients [9,10]. Since the eradication of smallpox and the subsequent cessation of the vaccination in the 1980s, the susceptibility of the human population for OPV spillover infections is increasing and, therefore, the risk of OPV adaption in humans [11].

Cowpox viruses are well characterized and known for a long time with references back to Edward Jenner, but their classification within the genus *Orthopoxvirus* is a matter of ongoing debate. The definition of *Cowpox virus* as a single species was historically based on host specificity and phenotypic properties, e.g., formation of hemorrhagic pocks on infected chorioallantoic membranes (CAM) of eggs and electron microscopy-mediated identification of A-type inclusion bodies (ATI) [12,13,14]. Genome characterization was initially done by restriction fragment length polymorphism (RFLP) [15] and currently by high-throughput sequencing (HTS) of whole genomes [16,17]. Recent phylogenetic investigations using full-length genomes demonstrated that “*Cowpox virus*” is rather a polyphyletic group than a single species [16,18].

Furthermore, to date the natural reservoir of CPXV has not been clearly identified. Rodents, especially the common vole (*Microtus arvalis*), the field vole (*Microtus agrestis*), and the bank vole (*Myodes glareolus*), are thought to act as natural reservoir hosts of CPXV [19,20]. Voles belong to the order Rodentia, family Cricetidae, subfamily Arvicolinae that is further divided into several tribes including tribe *Myodini* with genus *Myodes* (including the bank vole) and tribe *Arvicolini* with genus *Microtus* (including common vole and field vole) [21]. In contrast, mice and rats belong to the same order, Rodentia, but to a different family, Muridae. The bank vole is one of the most abundant rodent species in Europe, detected mostly in forest habitats. It is found in most parts of the Western Palearctic region from Spain and Great Britain in the west up to Siberia in the east [22]. Infections with CPXV or other OPV were confirmed in these vole species using serological and PCR analyses [5,19,20,23,24,25,26,27,28,29,30,31,32,33] (Fischer et al., submitted). Furthermore, OPV-reactive antibodies were detected in other rodent species like wood mouse (*Apodemus sylvaticus*), yellow-necked mouse (*Apodemus flavicollis*), striped field mouse (*Apodemus agrarius*), and even in shrews like the common shrew (*Sorex araneus*) [16,20,29,31,32,34,35,36]. CPXV isolates originating from natural reservoirs are rare, currently only isolates originating from common voles have been described [16,20,37]. Animal experiments proved the reservoir competence of common voles; inoculation with a common vole-derived CPXV strain resulted in an asymptomatic infection with virus shedding [37]. In contrast, bank voles seem to be resistant to experimental infection with strains derived from common vole, rat, cat, and human, questioning the reservoir competence of bank voles [38]. Currently no bank vole-derived CPXV isolate was reported, which might be used for an experimental proof of the reservoir competence of this vole species.

Here, we describe a qPCR-based CPXV-screening of bank voles collected in Thuringia, Germany, to gain new insights into the role of bank voles as potential reservoir of CPXV. A bank vole-derived CPXV strain was isolated, sequenced, and further characterized in vitro.

## 2. Materials and Methods

### 2.1. Rodent Trapping

**Figure 1:**
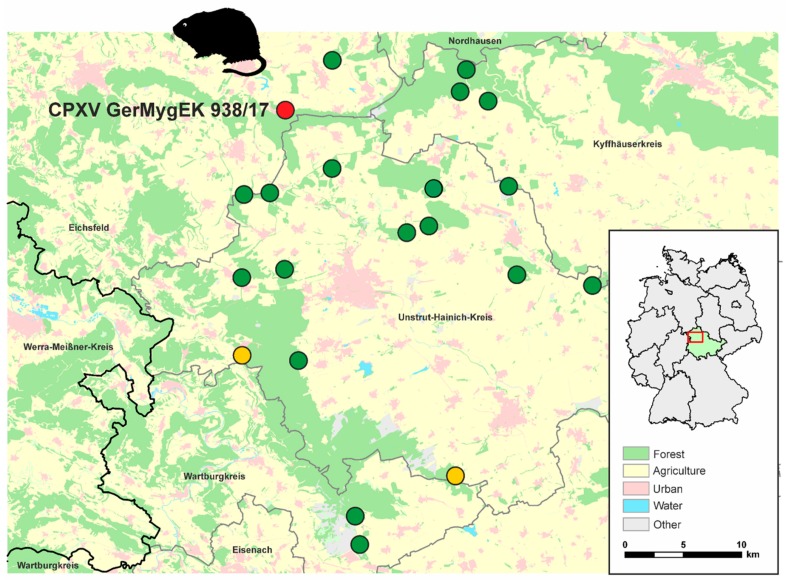
Bank vole trapping locations in Thuringia, Germany, during spring, summer, and fall, 2017 and 2018 (circles). The inset map of Germany shows Thuringia highlighted in green and the trapping area “Thüringer Becken” marked by a red frame. Yellow dots mark locations where *Cowpox virus* (CPXV)-DNA positive voles were sampled, and a red dot marks the trapping position of the bank vole from which the CPXV strain GerMygEK 938/17 was isolated. Green dots represent locations where only negative bank voles were sampled.

During spring, summer, and fall of the years 2017 and 2018, bank voles were collected by snap-trapping at 21 forest locations within Thuringia (“Thüringer Becken”), Germany (Figure 1 and Table S1) [39]. All procedures involving animals were conducted according to relevant legislation and by permission of the responsible authority in Thuringia (permit 22-2684-04-15-105/16, 13/04/2017). All collected voles were frozen at −20 °C until necropsy. During dissection, species, body size, weight, and sex were recorded. Nasal septum and kidney tissues were taken and stored at −20 °C until nucleic acid extraction.

For molecular confirmation of the rodent species, DNA was extracted from kidney tissue (Tissue DNA Kit, Roboklon, Berlin, Germany). Subsequently, a cytochrome *b* specific PCR was performed [40], PCR products were sequenced and compared to GenBank entries using Nucleotide Basic Local Alignment Search Tool (BLASTn)-based analysis.

### 2.2. OPV DNA Screening 

OPV DNA screening was based on nasal septum samples, as the nasal septum has been shown to be better suitable for OPV detection than other internal organs [37]. Nose septum samples were transferred into reaction tubes with 1 mL Eagle’s minimal essential medium (MEM; Biochrom GmbH, Berlin, Germany) supplemented with 10% fetal calf serum (FCS, Biochrom GmbH), antibiotics (1% penicillin-streptomycin, Biochrom GmbH) and stainless steel beads (5 mm in diameter, TIS Wälzkörpertechnologie GmbH, Gauting, Germany) for mechanic homogenization (TissueLyser II; Qiagen, Hilden, Germany). DNA extraction was done semi-automatically in a BioSprint 96 instrument (Qiagen) using the NucleoMag VET kit (Macherey-Nagel, Düren, Germany). The isolated DNA was analyzed using a quantitative polymerase chain reaction (qPCR) assay (QuantiTect Multiplex PCR NoROX Kit, Qiagen) targeting a 146 nucleotide (nt) region of the 14-kD protein-encoding (*A27L*) gene of CPXV [41].

### 2.3. Cell Lines and Virus Isolation

Virus isolation was performed with all CPXV DNA-positive nasal septum samples. Hence, overnight cultures of Vero76 cells (Collection of Cell Lines in Veterinary Medicine CCLV, CCLV-RIE 0228, Friedrich-Loeffler-Institut, Greifswald-Insel Riems, Germany), were inoculated with 100 µL of the homogenized tissue material and kept at 37 °C under a 5% CO_2_ atmosphere.

Vero76 cells were grown and maintained in MEM supplemented with 10% FCS containing antibiotics (1% Enrofloxacin; Bayer, Leverkusen, Germany; 0.2% Amphotericin/Gentamicin; Thermo Fisher Scientific Inc., Schwerte, Germany; 0.5% Linomycin; WDT, Garbsen, Germany).

Inoculated cells were passaged until a cytopathic effect (CPE) was observed, and virus was collected in Tris-EDTA buffer. For virus detection and characterization, a qPCR [20] and whole genome sequencing was performed. The obtained virus isolate was designated GerMygEK 938/17 indicating the country of origin, Germany; the animal species, *Myodes glareolus;* the trapping location, Eichsfelder Kessel; the individual number (938) and the year of trapping, 2017.

### 2.4. Sequencing, Genome Assembly, and Annotation of the CPXV Isolate

Viral DNA was extracted from CPXV positive cell culture using the MasterPure™. Complete DNA and RNA Purification Kit (Lucigen Simplifying Genomics, Middleton, WI, USA) according to the manufacturer’s instruction. The DNA preparation was submitted to Eurofins GATC Biotech (Konstanz, Germany) for HTS. In total, 5 million paired-end reads with a read length of 150 base pairs (bp) were obtained using the Illumina HiSeq 4000 platform. The reads were quality and adapter trimmed using the 454 Sequencing System Software (version 3.0; Roche, Mannheim, Germany) along with appropriate Illumina specific adapter sequences. Subsequently, the trimmed reads were mapped to a non-redundant version (one copy of the ITR deleted) of the genome of CPXV strain Ger2010MKY (lineage tentatively named “CPXV-like 3” [16]; LT896721.1) using Bowtie2 (version 2.3.4.3; [42]), and only mapped reads were further used for de novo assembly using 454 Sequencing System Software (version 3.0; Roche). The resulting contigs were subsequently used as reference sequences for an additional round of mapping and de novo assembly. The iterative process was repeated five times, and the resulting contigs were then arranged to resemble the entire CPXV genome. The resulting full-length CPXV sequence was annotated analogous to the nomenclature of the CPXV Brighton Red (BR) reference strain (AF482758) as described elsewhere [37] as well as to the closely related CPXV strain Ger 2010 MKY (LT896721) [43]. Additional tentative open reading frames (ORF) were numbered as follows: gCPXV0XXX for genes and pCPXV0XXX for proteins [37].

### 2.5. Phylogenetic Analysis

An alignment of representative OPV complete genomes (Supplementary Table S1) was constructed using MAFFT within Geneious (version 11.1.5; https://www.geneious.com; Biomatters Limited, Auckland, New Zealand). Poorly aligned regions were removed by using trimAl (version 1.2; [44]) as implemented in Phylemon (version 2.0; [45]). A phylogenetic tree was constructed using IQ-TREE (version 1.6.10; [46]), visualized using FigTree (version 1.4.3; [47]) and clades were compressed using MEGA (version 7.0.14; [48]). The similarity plot was constructed with a sequence alignment of CPXV GerMygEK 938/17 with reference strains CPXV Ger2010MKY (LT896721), OPV Abatino (MH816996) and ECTV Moscow (NC_004105) aligned with MAFFT within Geneious. The complete alignment and selected gene alignments were used under RStudio (version 1.1.463, [49]) showing the identity of the sequences. In addition, potential recombinant sequences were parsed using the exact algorithms established before [16]. In brief, the analysis was based on the Jukes–Cantor substitution model using a sliding window of 5000 nt, a step size of 100 nt, and bootstrap support by 100 replicates.

### 2.6. Phenotypic Analysis Using Chorioallantoic Membrane Culture

Embryonated chicken eggs, obtained from the Friedrich-Loeffler-Institut, Insel Riems, were incubated at 37 °C and a relative humidity of 50% in an incubator for 10 to 13 days. Inoculation onto the chorioallantoic membrane (CAM) was performed as described in [50]. In brief, a hole was abraded in the eggshell using a grinder (Dremel, Racine, WI, USA), and CAMs were inoculated with 10^5^ tissue culture infectious dose 50 (TCID_50_) of virus in 100 µL phosphate-buffered saline (PBS) or with the same volume of PBS as mock control. Inoculated eggs were sealed with paraffin wax and incubated at 37 °C for 72 h without moving. CAMs were harvested after chilling all eggs for at least 2 h at 4 °C, washed at least three times with PBS, and photographed immediately. CAM were inoculated with ECTV strain US#4619, CPXV strain RatPox09 or the novel isolate CPXV GerMygEK 938/17. 

### 2.7. Virus Replication Kinetics in Different Cell Lines

To analyze the replication kinetics, the novel bank vole-derived strain GerMygEK 938/17, the common vole-derived strain FM2292 [37], and the commonly used laboratory strain Brighton Red (BR) [12] were tested in overnight cell culture. A multiplicity of infection (MOI) of 0.01 and 3 was used in (A) a bank vole-derived kidney cell line (BVK168, CCLV-RIE 1313; [51]) and (B) a common vole-derived kidney cell line (FMN-R, CCLV-RIE 1102; [52]) as well as on (C) Vero76 cells as a reference cell-line for CPXV growth. After 60 min at 37 °C, the inoculated cell cultures were washed three times with PBS, and fresh MEM supplemented with 10% FCS was added. Samples were collected at six time points post inoculation (0, 6, 12, 24, 48, and 72 h post inoculation (hpi)) including two biological replicates. Virus titers were determined by endpoint dilution assay and calculated as TCID_50_ mL^−1^ using the Spearman–Kärber algorithm [53,54].

### 2.8. Data Availability

The annotated full-length genome sequence of the bank vole-derived CPXV strain GerMygEK 938/17 and sample information was uploaded to the European Nucleotide Archive (ENA) and made publicly available under project accession PRJEB32300.

## 3. Results

### 3.1. Rodent Trapping and Detection of OPV DNA

A total of 533 bank voles were collected during 2017 and 2018 at 21 sites in Thuringia (Figure 1); for 509 carcasses nasal septum samples were available. OPV DNA was detected by qPCR in nasal septum samples of five out of 509 (0.98%) bank voles. Virus DNA-positive bank voles originated from three trapping areas and were captured in spring (*n* = 4, two females and two males) or summer (*n* = 1, male), in 2017. In spring, 2017, three of these positive voles originated from the same trapping area: Eichsfelder-Kessel. The C_q_-values of the individual samples varied between 21 and 38 (see Supplementary Table S2). 

### 3.2. Virus Isolation, Sequence Determination, and Genome Characterization

Virus isolation in Vero76 cells was successful for one out of the five OPV DNA-positive bank vole septum samples. After three passages, the novel CPXV strain GerMygEK 938/17 showed a titer of 10^6^ TCID_50_ mL^−1^ on Vero76 cells. HTS resulted in the determination of the complete genome sequence of CPXV GerMygEK 938/17 with 220,822 bp. Two contigs resulted from the assembly. One contig (206,524 nt) resembled the unique core of the genome, and the second contig resembled the terminal tandem repeat (7190 nt). Contig junctions were confirmed by manual inspection of the overlapping reads between the contigs. The mean sequence depth of the unique region was 750 and that of the terminal repeat region 1525. 

### 3.3. Phylogenetic Analysis

**Figure 2:**
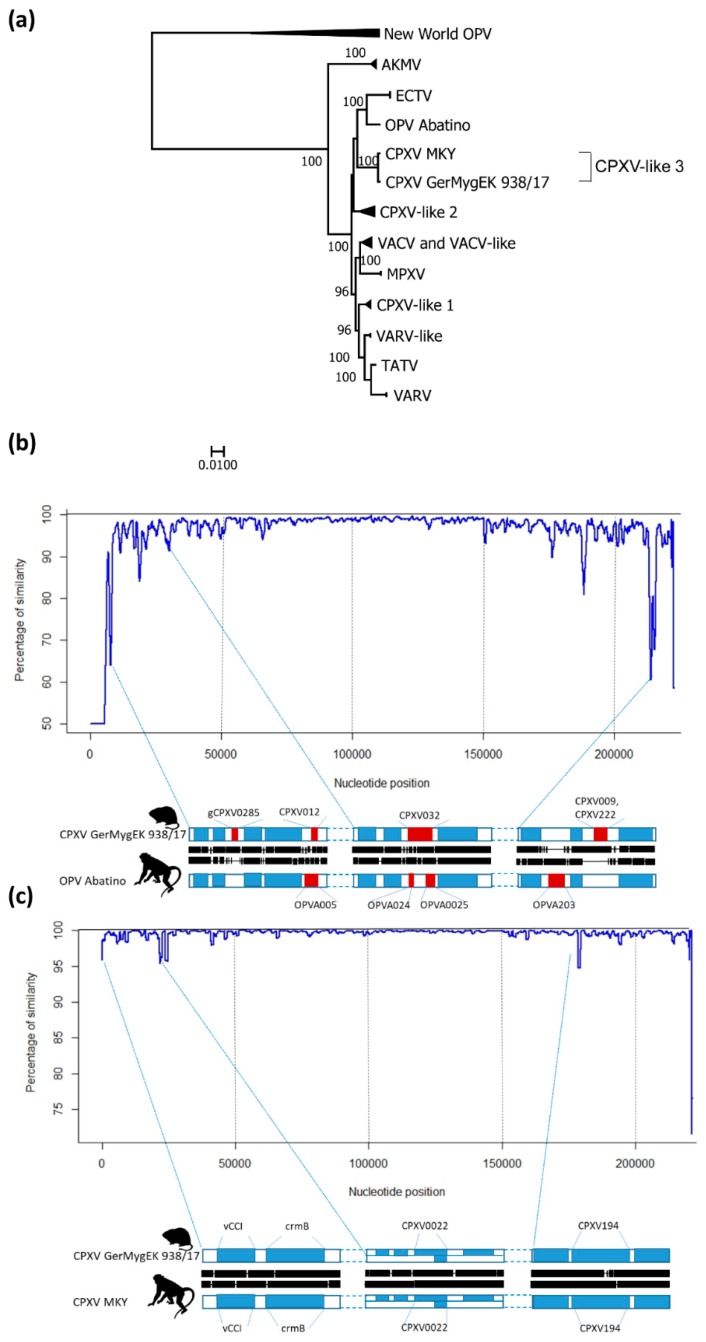
Phylogenetic analysis and sequence similarity analyses of CPXV GerMygEK 938/17. (**a**) Phylogenetic tree using New World orthopoxviruses as outgroup. Clades where named after Franke et al., 2017 [16]. Only bootstrap supporting values over 70 are given at the supported nodes. Black triangles indicate compressed branches. (**b**) Similarity plot showing the sequence identity between CPXV GerMygEK 938/17 (query sequence, black line) to the reference sequences OPV Abatino (MH816996, blue line) along their full genomes. Three regions exhibiting prominent differences are detailed below as a zoom-in visualization. Gaps between black lines indicate gaps in the DNA sequence. (**c**) Similarity plot showing the sequence identity between CPXV GerMygEK 938/17 (query sequence, black line) to the reference sequences CPXV MKY (LT896721, blue line) along their full genomes. Alignment details of three genomic parts are depicted below. Open reading frames (ORFs) that are conserved in both genomes are colored in blue, while ORFs that are present in either one of the two genomes are colored in red. White areas represent intergenic regions.

Comparative analysis of the full-length sequence of CPXV GerMygEK 938/17 showed the highest nucleotide sequence identity (99.2%) to a virus isolated from a cotton-top tamarin (*Saguinus oedipus*) in Germany 2010 (CPXV Ger2010MKY, GenBank accession number LT896721) [43] (Figure 2a). CPXV GerMygEK 938/17 and CPXV Ger2010MKY (CPXV MKY) sequences form a separate cluster provisionally named “CPXV-like 3” clade [16] in close proximity to the OPV isolate Abatino from Italy (MH816996) pairwise identity 93.8%, which was referred to as ECTV-like [55,56] and ECTV strains (pairwise identity ECTV strain Moscow 85.3%). Similarity plot analysis showed areas of high sequence variation between CPXV GerMygEK 938/17 and OPV Abatino (Figure 2b), while comparison to CPXV MKY indicated only small variations (Figure 2c). Analysis of the CPXV GerMygEK 938/17 sequence using a bootscan analysis revealed a similar pattern like the one established for CPXV Ger2010MKY [16] (data available upon request). We interpret CPXV GerMygEK 938/17 as second member of the separated lineage “CPXV-like 3”. 

### 3.4. Phenotypic Characterization on the CAM 

**Figure 3:**
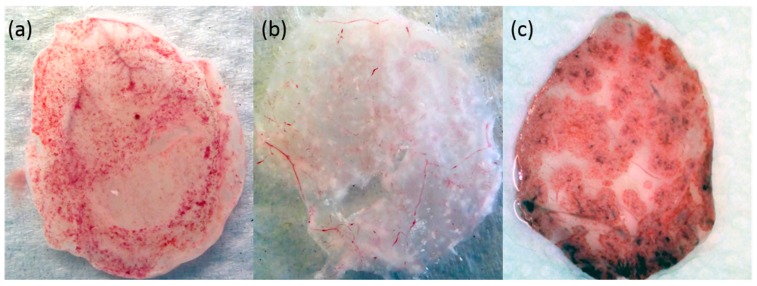
Lesions detected on chorioallantoic membranes (CAM) after inoculation with CPXV GerMygEK 938/17 (**a**), *Ectromelia virus* US#4619 (**b**) and RatPox09 (**c**). Lesions were photographed after 72 h incubation at 37 °C.

The CAM of chicken eggs showed CPXV-specific hemorrhagic pocks after inoculation with CPXV GerMygEK 938/17 comparable to the CPXV RatPox09 control (VARV-like clade) inoculation (Figure 3a,c). In contrast, white pock lesions on the CAM were detected after inoculation using ECTV (Figure 3b).

### 3.5. Virus Replication Kinetics in Different Cell Lines 

Isolates CPXV GerMygEK 938/17, CPXV FM2292, and BR were compared using single and multi-step growth kinetics in Vero76 and two vole cell lines (see Supplementary Figure S1). All three virus strains showed some level of replication independent of the cell line used. The highest virus titers regardless of the isolates were detectable on Vero76 cells. Here, the replication kinetics of all three CPXV strains were similar. While, the novel strain GerMygEK 938/17 clearly displayed an impaired replication capacity on both vole-derived cell lines, the virus is generally able to establish replication cycles, as passaging of the virus in these cells is possible (data available upon request). However, titers of CPXV GerMygEK 938/17 achieved on vole-derived cell lines at 48 h were in the range of the inoculated titers (see Supplementary Figure S1). Interestingly, the lowest titers were achieved on BVK168 cells derived from bank vole kidney tissue for all used viruses. 

## 4. Discussion

We detected CPXV-specific DNA in five out of 509 tested bank voles (0.98%) from Thuringia, Germany. The nasal septum sample from one of these DNA-positive bank voles was successfully used to isolate a novel CPXV strain. To our knowledge, this is the first description of a bank vole-derived CPXV isolate. 

The here observed CPXV DNA prevalence of 0.98% in bank voles is comparable to other studies that detected 0.19 up to 1.33% [31] (Fischer et al., submitted). OPV infections in bank voles—as detected by antibodies and/or DNA—were reported in different Eurasian countries: Belgium [28], Buryatia [31], England [19,23,24,25,26,27], Hungary [32], Norway [29,30], and Finland [31,33]. In line with our data, it seems that active CPXV infections in bank voles are rare. Therefore, virus persistence in endemic regions might be mediated by a high stability outside the host, as described for other OPV [34], rather than by a high number or proportion of infected voles. Unlike the common vole, which may be subject to infection with different CPXV strains resulting in seroconversion, virus shedding, and clinical signs, bank voles seem to be resistant, showing neither virus shedding nor clinical symptoms and exhibiting a low rate of seroconversion [37,38,57]. Thus, it is not clear whether bank voles are solitary maintenance/reservoir hosts for CPXV, or if they are only the reservoir for a special type of CPXV. In vitro testing of CPXV GerMygEK 938/17 as well as CPXV FM2292, a common vole isolate, resulted in infection of bank and common vole-derived cell lines regardless of the used CPXV-isolate. Interestingly, viral titers generated from infection of Vero76 cells were comparable and of highest level. Using bank vole and common vole-derived kidney cell lines, CPXV GerMygEK 938/17 exhibited at least tenfold-reduced viral titers at 24 h and at later time points. Probably interferon-related responses are responsible for this effect. In addition, vole cells of other tissue origins might be able to assist CPXV replication more potently.

Infection of CAM using the new bank vole CPXV strain GerMygEK 938/17 resulted in hemorrhagic pock formation. This was described as CPXV-specific phenotype in contrast to non-hemorrhagic “white” pocks seen after ECTV inoculation of CAM cultures [12,13,14].

Interestingly the CPXV GerMygEK 938/17 sequence clustered close to ECTV, a virus causing white pocks lesions and not able to infect bank voles even with high titer inoculation [58]. ECTV itself has so far only been detected in laboratory colonies of house mice (*Mus musculus*) except for the human strain ERP [59]. In comparison to CPXV, ECTV comprises a shortened genome and a reduced number of genes, whereby many of the lost genes are associated with host interaction [60,61]. It seems likely that ECTV originated from a CPXV-like ancestor virus and adapted to house mice together with the loss of viral genes. The loss of hemorrhagic phenotype-related genes resulted in the white pox lesion phenotype on CAM [62].

The bank vole-derived isolate clustered closest to strain CPXV Ger2010MKY isolated from a cotton-top tamarin from a zoo in Thuringia [43]. Therefore, it seems likely that cotton-top tamarin infection was mediated by bank voles. While rodents, including bank voles, were trapped in the surroundings of the zoo, none were tested positive for CPXV [43]. This might be explained by the low number of tested rodents (*n* = 23) compared to the current study. The closest branch to CPXV GerMygEK 938/17 and CPXV Ger2010MKY consists of OPV Abatino isolate from an Italian Tonkean macaque (*Macaca tonkeana*). This strain is very similar to another OPV-derived sequence from an Italian cat [55,56,63]. During the Italian macaque outbreak, trapped rodents were tested negative for OPV-reactive antibodies [55]. 

Overall, it seems reasonable to assume that bank voles might also be the source of the CPXV spillover cases in Italy. However, this would need further testing as during the outbreak in Italy no bank voles were trapped, and it is also important to include higher numbers of bank voles in future monitoring studies since the expected prevalence of viremic bank voles is low.

The observed genetic distance of bank vole-derived CPXV GerMygEK 938/17 to common vole-derived isolates CPXV FM2292 and CPXV Ger/2007/vole, both from Baden-Wuerttemberg (CPXV-like 2 clade), and CPXV FMEimka from Saxony (CPXV-like 1 clade), implies that at least three different vole-associated CPXV lineages exist in Germany [16,20]. This finding may suggest that CPXV in general is maintained by multiple small mammal species, mainly voles, rather than by one specific host [4,16,18]. Moreover, highlighted by the fact that bank voles were not susceptible for productive infection with a common vole-derived CPXV FM2292 strain [38], but replicating virus CPXV GerMygEK 938/17 could be isolated, vole species-dependent CPXVs clades might be the reason for high seroprevalences detected in bank voles throughout Europe. For a definite answer, experimental inoculations of different vole species using different CPXV-isolates would be necessary. 

Currently, CPXV isolates, especially isolates from voles and other wild rodents are out-numbered by isolates from accidental hosts like cats, alpacas, or zoo animals. Therefore, a Europe-wide screening of rodent populations for CPXV-infected individuals would help to better understand CPXV-reservoir host relationships and the role of voles for CPXV transmission. In addition, virus isolation combined with whole-genome sequencing is essential to gain deeper insights into CPXV phylogeny, distribution of CPXV clades, as well as OPV evolution in general. 

## 5. Conclusions

Here we provide the first description and in vitro characterization of a bank vole-derived CPXV-isolate. Interestingly, the analyzed whole-genome sequence revealed a clustering of this isolate next to ECTV, the orthopox virus of mice, also phenotypically both viruses differ drastically in the CAM system. With the first direct evidence of replication competent CPXV in bank voles and together with the reports about seropositive bank voles, bank voles have also to be considered as a reservoir species for CPXV. Due to limited sample numbers and the localized screening, the presented results are biased. Nevertheless, bank voles have a very wide distribution area in Eurasia and the detection of related sequences in Italy suggests a possibly similar wide distribution of bank vole-derived CPXVs. We therefore would like to encourage the monitoring of further bank vole samples as well as other small mammals in order to improve the data basis about the occurrence, genetic variability, and reservoir hosts of CPXV. 

## Supplementary Materials

The following supplementary figure and tables are available online at https://www.mdpi.com/1999-4915/11/11/1075/s1. Figure S1: Virus replication kinetics of vole derived-CPXV on different cell lines. Table S1: Individual information on the bank voles (*M. glareolus*) tested positive for orthopoxvirus (OPV) DNA. Table S2: List of orthopoxvirus (OPV) sequences and classification into several clades.
