# Supplementary material for: Molecular Detection and Characterization of the First Cowpox Virus Isolate Derived from a Bank Vole
Source: Viruses. 2019 Nov 18;11(11):1075. doi: 10.3390/v11111075 (PMC6893522; doi:10.3390/v11111075)
Supplement: Supplementary file 1 [file viruses-11-01075-s001.zip › Table S1 and Table S2.pdf]

Supplementary Table 1. List of used Orthopox virus sequences and classification into species and clades: *Ectromelia virus* (ECTV), *Monkeypox virus* (MKXV), New World Orthopoxvirus (New World OPV), *Variola virus* (VARV), *Vaccinia virus* (VACV), *Taterapox virus* (TATV), Cowpox virus like 1 (CPXV-like 1), Cowpox virus like 2 (CPXV-like 2) , Cowpox virus vaccinia virus like (VACV like), and Cowpox virus variola virus like (VARV-like) according to Franke et al.,2017 [1]. Additional clades introduced due to phylogenetic tree positions includes Akhmeta virus (AKMV), and Cowpox virus Ectromelia virus like (ECTV-like).

Accession number	Name	Species / Clade
AF012825	Ectromelia virus strain Moscow	ECTV
JQ410350	Ectromelia virus ERPV culture-collection ATCC:VR-1431	ECTV
KJ563295	Ectromelia virus Naval	ECTV
MH816996	Orthopoxvirus Abatino	CPXV ECTV-like
DQ011154	Monkeypox virus strain Congo_2003_358	MKXV
DQ011155	Monkeypox virus strain Zaire_1979-005	MKXV
NC_027213	Raccoonpox virus	New World OPV
NC_031033	Volepox virus strain CA	New World OPV
NC_031038	Skunkpox virus strain WA	New World OPV
DQ437584	Variola virus strain Germany 1958 Heidelberg	VARV
DQ441429	Variola virus strain Japan 1946 (Yamada MS-2(A) Tokyo)	VARV
KX061501	Vaccinia virus strain Lister	VACV
DQ439815	Vaccinia virus strain DUKE	VACV
DQ792504	Horsepox virus isolate MNR-76	VACV
NC_008291	Taterapox virus	TATV
KC813491	Cowpox virus strain BeaBer04/1	CPXV-like 1
LT896731	Cowpox virus isolate Ger/2015/Cat4 genome assembly	CPXV-like 1
LT993228	Cowpox virus isolate Ger/2017/common vole FMEimka	CPXV-like 1
LT993232	Cowpox virus isolate Ger/2015/Human2	CPXV-like 1
AF482758	Cowpox virus strain Brighton Red	CPXV-like 2
DQ437593	Cowpox virus strain Germany 91-3	CPXV-like 2

LN864566	Cowpox virus FM2292	CPXV-like 2
LT896722	Cowpox virus isolate Ger/2007/Vole	CPXV-like 2
HQ407377	Cowpox virus strain Austria 1999	CPXV VACV-like
HQ420893	Cowpox virus strain Finland_2000_MAN	CPXV VACV-like
KC813512	Cowpox virus strain HumKre08/1	CPXV VARV-like
LN864565	Cowpox virus Ratpox09	CPXV VARV-like
LT896721	Cowpox virus isolate Ger 2010 MKY	CPXV ECTV-like
MH607141	Akhmeta virus isolate Akhmeta_2013-88	AKMV
MH607142	Akhmeta virus isolate Akhmeta_2013-85	AKMV
MH607143	Akhmeta virus isolate Vani_2010	AKMV

[1] Franke A, Pfaff F, Jenckel M, Hoffmann B, Hoper D, Antwerpen M, Meyer H, Beer M, Hoffmann D (2017) Classification of Cowpox Viruses into Several Distinct Clades and Identification of a Novel Lineage. Viruses 9 (6). doi:10.3390/v9060142

Supplementary Table 2. Individual information on bank voles (*M. glareolus*) tested positive for orthopoxvirus genome in this study. Trapping areas indicate the area of capture shown in Fig.1. Organ material from the bank vole written in bold was used to generate CPXV GerMygEK 938/17.

Number	Date of trapping	Season	Species	Sex	Reproductive Status	weigth (g)	Head-to-torso-length (cm)	Tail-length (cm)	Ct-value
KS17/920	20.04.2017	Spring	*M. glareolus*	male		16	7.5	3.5	29
KS17/934	21.04.2017	Spring	*M. glareolus*	male	descended testis	30	8.5	4	26
**KS17/938**	**21.04.2017**	**Spring**	***M. glareolus***	**female**		**15**	**7.5**	**3.5**	**21**
KS17/973	10.05.2017	Spring	*M. glareolus*	female		17	8	3	34
KS17/1472	17.08.2017	Summer	*M. glareolus*	male		7	7	4	38
